# Analysis of the Frictional Performance of AW-5251 Aluminium Alloy Sheets Using the Random Forest Machine Learning Algorithm and Multilayer Perceptron

**DOI:** 10.3390/ma16155207

**Published:** 2023-07-25

**Authors:** Tomasz Trzepieciński, Sherwan Mohammed Najm, Omar Maghawry Ibrahim, Marek Kowalik

**Affiliations:** 1 Department of Manufacturing Processes and Production Engineering, Faculty of Mechanical Engineering and Aeronautics, Rzeszow University of Technology, al. Powst. Warszawy 8, 35-959 Rzeszów, Poland; 2 Kirkuk Technical Institute, Northern Technical University, 36001 Kirkuk, Iraq; sherwan.mohammed@gpk.bme.hu; 3 Department of Manufacturing Science and Engineering, Budapest University of Technology and Economics, Műegyetemrkp 3, H-1111 Budapest, Hungary; 4 Plant Production Department, Arid Land Cultivation Research Institute, City of Scientific Research and Technological Applications SRTA-City, Borg Al-Arab 21934, Egypt; oibrahim@srtacity.sci.eg; 5 Faculty of Mechanical Engineering, Kazimierz Pulaski University of Technology and Humanities in Radom, 54 Stasieckiego Street, 26-600 Radom, Poland; m.kowalik@uthrad.pl

**Keywords:** aluminium alloys, coefficient of friction, friction, sheet metal forming, tribology

## Abstract

This paper is devoted to the determination of the coefficient of friction (COF) in the drawbead region in metal forming processes. As the test material, AW-5251 aluminium alloys sheets fabricated under various hardening conditions (AW-5251-O, AW-5251-H14, AW-5251-H16 and AW-5251H22) were used. The sheets were tested using a drawbead simulator with different countersample roughness and different orientations of the specimens in relation to the sheet rolling direction. A drawbead simulator was designed to model the friction conditions when the sheet metal passed through the drawbead in sheet metal forming. The experimental tests were carried out under conditions of dry friction and lubrication of the sheet metal surfaces with three lubricants: machine oil, hydraulic oil, and engine oil. Based on the results of the experimental tests, the value of the COF was determined. The Random Forest (RF) machine learning algorithm and artificial neural networks (ANNs) were used to identify the parameters affecting the COF. The R statistical package software version 4.1.0 was used for running the RF model and neural network. The relative importance of the inputs was analysed using 12 different activation functions in ANNs and nine different loss functions in the RF. Based on the experimental tests, it was concluded that the COF for samples cut along the sheet rolling direction was greater than for samples cut in the transverse direction. However, the COF’s most relevant input was oil viscosity (0.59), followed by the average counter sample roughness Ra (0.30) and the yield stress R_p0.2_ and strength coefficient K (0.05 and 0.06, respectively). The hard sigmoid activation function had the poorest R^2^ (0.25) and nRMSE (0.30). The ideal run was found after training and testing the RF model (R^2^ = 0.90 ± 0.028). Ra values greater than 1.1 and R_p0.2_ values between 105 and 190 resulted in a decreased COF. The COF values dropped to 9–35 for viscosity and 105–190 for R_p0.2_, with a gap between 110 and 130 when the oil viscosity was added. The COF was low when the oil viscosity was 9–35, and the Ra was 0.95–1.25. The interaction between K and the other inputs, which produces a relatively limited range of reduced COF values, was the least relevant. The COF was reduced by setting the R_p0.2_ between 105 and 190, the Ra between 0.95 and 1.25, and the oil viscosity between 9 and 35.

## 1. Introduction

**Figure 1:**
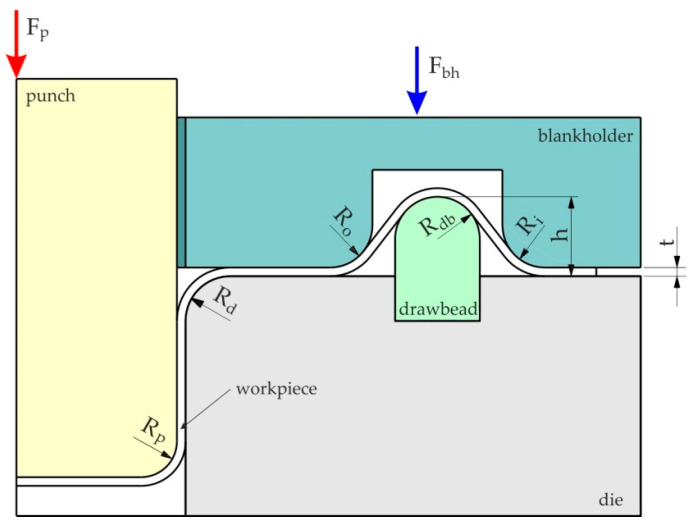
Deformation of the sheet metal in the drawbead region.

There is a growing interest in the automotive industry in sheets made of non-ferrous metal alloys, which, with strength equal to steel, are characterised by lower weight [1,2]. The production of sheet metal drawpieces characterised by low drawability is associated with the need to overcome the many problems related to elastic deformations of products and the limited formability [3,4]. Moreover, some lightweight alloys, for example, aluminium alloys, are prone to galling. During sheet metal forming, there are many phenomena and factors that determine the final shape and properties of the product. In obtaining products of appropriate quality, one of the key phenomena is that of friction occurring at the interface between the surfaces of the tool and the deformed material [5,6]. Currently, the primary method of reducing friction is to use lubrication [7]. Due to the occurrence of various friction conditions in the deep-drawing process, many tests have been developed to model friction, for example, the strip drawing test [8], the bending under tension test [9] and the drawbead test [10]. When forming drawpieces with a complex shape, it may be necessary to use drawbeads which direct the flow of the material by introducing tensile stresses in the sheet material. The drawbeads are protrusions on the die surface (Figure 1) through which the flange of the shaped drawpiece flows [11]. The shape of the drawbead is adapted to the thickness and plastic properties of the sheet material and controls the flow of the sheet metal into the die, but at the same time not prematurely stopping the flow of the material and breaking the drawpiece. Knowledge of the coefficient of friction (COF) occurring at the drawbead is necessary for designing the stamping tool and determining the appropriate boundary conditions in finite element-based numerical simulations of the sheet metal forming process.

Over the years, experimental methods have been developed to determine the value of the COF on the drawbead and the effect of material properties, sheet thickness, and the geometry of the drawbead on the resistance to friction and the restraining force. Firstly, Nine [12] developed a roller drawbead fixture to determine the COF. In this test, while pulling the sheet metal strip over the fixed and freely rotatable rollers, the pulling force and the clamping force are measured, so that it is possible to separate the sheet metal deformation resistance from the resistance to friction. Green [13] experimentally determined in situ bending radii in a strip while it was being pulled through a drawbead. The bead penetration had an effect on the amount of bead wrap. As the penetration increased, the wrap angle increased. Nanayakkara and Hodgson [14] modified the method proposed by Green [13] and determined the drawbead contacts with variable bead penetration. They concluded that the actual COF is a function of the angle of contact of the sheet metal with the drawbead. They also conducted finite element-based computations to study the deformation mechanics during the flow of the sheet metal through the drawbead. Samuel [15] established the finite element method (FEM) model for the investigation of blank-holding conditions and the COF on the deformation of the material which will be passed through the drawbead. Livatyali et al. [16] proposed a tribometer for testing the friction performance when the sheet metal passes through the drawbead. This tribometer is mounted in the holders of a universal testing machine, enabling the determination of only the value of the pulling force of the sheet metal. The depth of the drawbead is regulated by means of a double-acting pneumatic actuator. It was found that the main factor determining the value of the restraining force is the height of the drawbead. Smith et al. [17] developed a device for determining the pulling force of the sheet metal in a stamping die with a drawbead. When the sheet metal is pulled through the height-adjustable drawbead, the pulling force, the restraining force, and the displacement of the sheet are measured. The results of drawing strips of deep-drawing quality steel sheets confirmed quantitatively that the parameter that determines the value of the restraining force to the greatest extent is the geometry of the drawbead. Murali et al. [18] analysed the effect of drawbead geometry in the sheet metal drawing process using finite element analysis and experimental validation. The work-hardening phenomenon plays an important role in the restraining forces. A rectangular drawbead bends and unbends the sheet four times and strengthens the material more than a circular drawbead. Firat et al. derived an analytical model for predicting the restraining force [19] and proposed a numerical model to improve the contact and wrap angle in the drawbead region [20]. Thipprakmas [21,22] numerically analysed the forming process of SUS304 stainless steel with and without using drawbeads. The results showed that it is difficult to achieve a straight wall on both the ‘length’ and ‘width’ sides without the use of a drawbead. Smith et al. [17] developed a device for obtaining the pulling forces for drawbead tooling on inclined binder surfaces in the Oakland University Angle Draw Bead Simulation (OU-ADBS) test. For the deep-drawing steel sheets, bead penetrations, and bead geometries studied, the pulling force at a 20° binder angle ranged from 55% to 96% of that corresponding to a 0° binder angle. Bassoli et al. [23] developed a versatile drawbead simulator to measure the restraining force when deep drawing 6014-T4 aluminium alloy sheets. The authors investigated the effects of the geometry of the drawbead on the restraining forces. It was found that the restraining force normalised by the drawbead width increased with increasing values in the bead height-to-radius ratio. De Carvalho and Lukács [24] used friction shear stress values to detect those places on the workpiece that were exposed to tangential stress caused by contact with the tools and to verify the drawbeads. For the analysed region, the coulomb model was found to be more relevant than the pressure-dependent model for describing friction phenomena. Lo [25] investigated the restraining force generated by the draw bead under different settings for the draw bead radius. It was concluded that when a higher force was applied to the draw bead, a higher restraining force was generated. Gil et al. [26] proposed a model to identify the main variables affecting the uplift force in the drawbead region. They found that minor geometrical deviations in the drawbead geometry significantly affect the uplift force. Venema et al. [27] observed that the coating flakes transported by the sliding sheet tend to accumulate in the area of high contact pressure (the drawbead region). Zabala et al. [28] found a direct relationship between the COF, thinning and forming limit diagram. The lower the COF, the higher the draw-in of the material. In the draw bead region, the clamping force is about 85% of the restraining force. When the restraining force is created by friction only, the binder force needs to be five to seven times larger depending on COF [29]. 

The phenomenon of friction in sheet metal forming is the result of the simultaneous interaction of many parameters and phenomena such as normal pressure, the surface roughness of the sheet metal and dies, the temperature, the combination of workpiece-tool materials, and the sliding speed. In practice, it is very difficult to determine the influence of one parameter on the COF while omitting other parameters. Therefore, analytical modelling, analysis of variance, artificial neural networks (ANNs), machine learning, genetic algorithms, and advanced deep learning techniques are used. Among the mentioned methods, ANNs are the most widespread and their operation is understood well. In recent years, there has been an interest in artificial intelligence (AI) techniques for solving problems related to the phenomenon of friction, of which ANNs predominate. The performance of ANNs depends on many parameters, that is, the number of hidden layers [30], the training algorithm [31], the number of neurons in the hidden layers [32] and the quality of the input data [33], the neuron activation function [34], and the size of the dataset (a larger training database provides greater accuracy for ANNs). Najm et al. [35] used neural networks to identify the parameters influencing the COF of the deep-drawing sheets tested in the strip drawing test. Different methods of partitioning weight were employed for the expected COF to assess the individual feature’s relevance. Lemu et al. [36] developed mathematical models that describe the friction behaviour of brass sheets using multiple regression analysis and ANNs. They concluded that one advantage of ANNs is that they can be used to extrapolate the model’s function outside the range of the training set but this only has limited scope. Trzepieciński et al. [37] used backward elimination regression and multilayer ANNs to predict the COF of Ti-6Al-4V titanium alloy sheets based on the results of the strip drawing test. Different training methods, that is, the quasi-Newton, Levenberg–Marquardt, back propagation, and network architectures were considered. The highest value of the correlation coefficient and the minimum value of the round mean square error were found for a multilayer perceptron (MLP) trained using the quasi-Newton algorithm. Yang et al. [38] used generalised regression neural networks for predicting the COF of Cr_1−x_Al_x_C films deposited on high-speed steel substrates. It was found that compared to the conventional back propagation ANNs, generalised regression neural networks are more suitable for predicting the COF of Cr_1−x_Al_x_C films. Nasir et al. [39] applied ANNs for predicting the COF of multilayer chopped strand mat glass fibre-reinforced thermosetting polyester composites in three different orientations. Various training functions were used and it was found that single-layer ANNs showed a prediction accuracy of up to 90% when trained with the Levenberg–Marquardt function. The ANN prediction method has been used in several applications, such as friction [40], surface roughness [41], and wear [42]. The potential applications of ANNs in the field of tribology were provided in review papers by Frangu and Ripa [43] and Argatov [44]. Perspectives about the use of AI in tribology were also provided by Rosenkranz et al. [45].

There are many approaches in the literature investigating the friction performance of materials. However, to the best of the authors’ knowledge, the problem of determining the COF in the drawbead region using the Random Forest (RF) machine learning algorithm combined with ANNs has not been analysed so far. In this paper, a machine learning algorithm and ANNs were used to identify the parameters affecting the COF of AW-5251 aluminium alloy sheets in different temper designations, that is, O, H14, H16, and H22. The sheets were tested using a drawbead simulator under different friction conditions (dry friction, oil lubrication), the different surface roughness of the countersamples and different orientations of the samples in relation to the direction of the sheet rolling. Machine learning is a collection of algorithms focusing and on making precise predictions from data [46]. One of these algorithms is RF [47] which is considered a non-parametric supervised machine learning algorithm. It consists of decision trees where each tree is split by selecting random inputs to fit a random bootstrap sample of those inputs. Another algorithm of machine learning is ANNs which are considered a robust mathematical model and consist of interconnected simple processing units called neurons just like the biological neurons in humans. The neurons in ANNs are arranged in sets called layers. Each neuron in a given layer is connected to other neurons in the adjacent layers but not to neurons in the same layer. The connections among neurons are defined by what is known as connection strength or connection weight which are used in estimating the relative importance of the inputs. In the present study, RF and ANNs were used to explore the relationship between the yield stress R_p0.2_, the average roughness of the countersamples Ra, oil viscosity, and the strength coefficient K with the COF as well as estimating their relative importance.

## 2. Material and Methods

### 2.1. Test Material

AW-5251 aluminium alloy sheets in different temper states (Table 1) were used as the test materials. The chemical composition of the AW-5251 aluminium alloy according to the EN 573-3 [48] standard is listed in Table 2. The mechanical properties of the sheets in Table 3 were determined in a uniaxial tensile test for samples cut along and transversely to the direction of the sheet rolling. The uniaxial tensile tests were carried out at room temperature according to the EN ISO 6892-1 [49] standard. The basic mechanical properties were determined, that is, yield stress R_p0.2_, ultimate tensile stress R_m,_ and elongation A_50_. The parameters of work hardening were determined based on the approximation of the true-stress/true-strain curves using the power law hardening (or Ludwik/Holloman) equation σ_p_ = K·ε^n^, where σ_p_ = stress, K—strength coefficient, n—strain hardening exponent, ε—plastic strain. The basic surface roughness parameters of the tested sheets (Table 4) were determined using an Alicona Infinite-Focus instrument.

### 2.2. Experimental Setup

**Figure 2:**
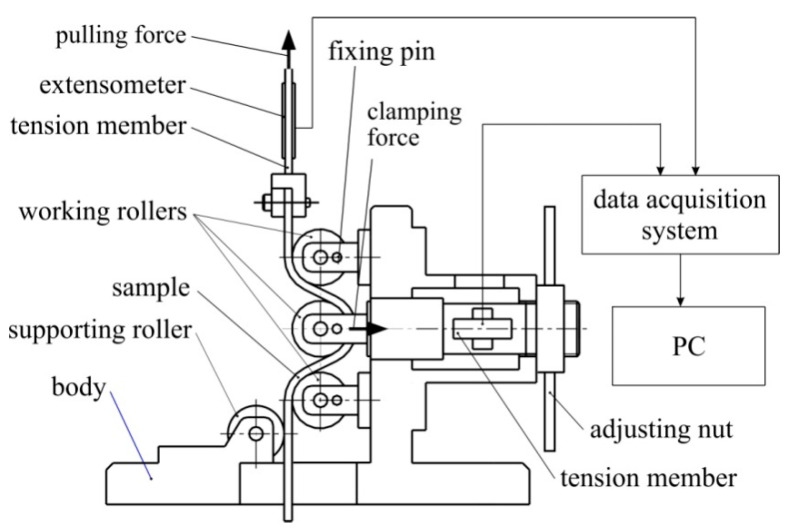
Drawbead simulator [10].

Tests to determine the COF in the drawbead region require the separation of the deformation resistance from the resistance to friction as the sheet metal passes through the drawbead. For this purpose, a four-roller tester simulating the sheet passing through the drawbead in sheet metal forming was developed (Figure 2). The tribotester’s equipment includes three sets of cylindrical countersamples made of cold-worked tool steel X165CrV12 with a diameter of 20 mm and an average roughness Ra of 0.32, 0.63 and 1.25 µm. Due to the rotating shape of the countersamples and the manufacturing technology, the average roughness was measured on the surface of the rollers, parallel to their axes. The device was mounted in the lower holder of a universal uniaxial tensile testing machine.

A sample in the form of a 20 mm wide and 200 mm long strip of sheet metal was placed between the lower and upper working countersamples and the middle working countersample. The upper end of the strip sample was then clamped into the tension member, which was then clamped into the upper fixture of the testing machine. The height of the drawbead was set by the appropriate horizontal displacement of the middle working countersample. The tests were carried out with a drawbead height of 20 mm. In this way, the axes of all three working rollers lay in one geometrical plane. The supporting roller was used to prevent the sample from bending due to elastic deformations of the sheet during the friction test. The value of the pulling and clamping forces was recorded using extensometers bonded to the tension members (Figure 2). The signals from the extensometers were transmitted to an 8-channel HBM amplifier, and then to a computer with software.

The determination of the coefficient of friction requires two tests: with freely rotatable rollers and with fixed rollers. During tests with freely rotatable rollers, the clamping force F_c_ and the pulling force F_p_ were registered. Similarly, during tests with fixed countersamples, the clamping force F_cf_ and the pulling force F_pf_ were registered. Then the value of the COF was calculated according to the relationship [14]:

(1) $$\mu =\frac{\sin\alpha }{2\alpha }\cdot \frac{F_{\mathrm{pf}}-F_{p}}{F_{\mathrm{cf}}}$$

where α is half of the centre roll wrap angle, α = 90°.

The friction tests were carried out using sheet strips cut along the rolling direction and at a transverse direction to that of the sheet rolling. The samples were drawn at a speed of 1 mm/s under conditions of dry friction and lubrication with three oils: L-AN 46 machine oil, SAE10W40 engine oil, and L-HL 32 hydraulic oil. The basic physicochemical properties of the test oils are shown in Table 5. Sheet metals in the as-received state were tested after cleaning their surfaces using acetone.

### 2.3. ANN Modelling

**Figure 3:**
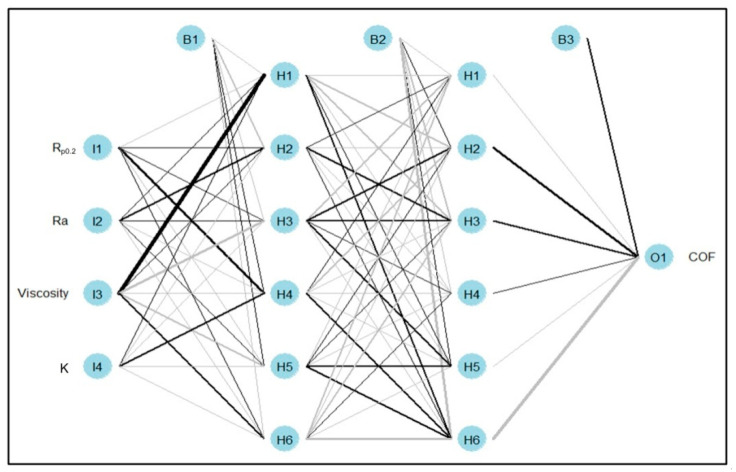
Structure of the multilayer perceptron used: I, H, B, and O stand for input, hidden, bias, and output neurons. Black colour connections indicate a negative effect while grey colour connections indicate a positive effect, the thickness of the connections indicates the degree or the strength of the effect.

In the present study, a four-layer feed-forward multilayer perceptron neural network (Figure 3) was used to study the relationship between the inputs (R_p0.2_, Ra, oil viscosity, and K) and the output (COF). The structure of the network consisted of one input layer with four neurons, two hidden layers with six neurons each, and one output layer with one neuron. A back propagation learning algorithm was used and 12 activation functions were compared based on the determination of the coefficient (R^2^) and the normalised root mean square of error (nRMSE). The 12 activation functions were *relu*, *gelu*, *softplus*, *swish*, *sigmoid*, *hard_sigmoid*, *elu*, *selu*, *leaky_relu*, *softsign*, *tanh*, and *linear*. To train the network, 80% of the data set was used and the other 20% was used for testing its performance. After training and testing the network, the connection weights algorithm [50,51] was used to calculate the relative importance of the inputs. It is worth mentioning that the output layer’s activation function was linear. The connection weights were generated using a Glorot uniform initializer to avoid gradient vanishing problems in the deep learning neural networks; the learning rate was 0.01, the momentum was 0.9, and the epochs were 350.

### 2.4. RF Model

The main reason for choosing the RF model in the present study is its good prediction performance [52]. Other reasons are that RF can be used for both regression and classification problems, it can handle high-dimensional data where both non-linear effects of inputs and the interactions among them exist, and it can be used to rank the inputs according to their relative importance. The RF model was tuned by adjusting its three tuning parameters to maximise its prediction capability. These three tuning parameters are the number of trees (*ntree*), the minimum number of data points at a leaf (*nodesize*), which is used for preventing overfitting, and the maximum number of randomly selected inputs (*mtry*) to be considered at each split node [53,54]. The best performance was achieved by using low values of the minimum node size and high values of the number of randomly selected inputs. Partitioning the dataset into training and testing subsets significantly affects prediction accuracy and training performance [55]. Inappropriate subsets have a negative effect on performance metrics. On the other hand, Shahin [56] argued that there is no evident relationship between the splitting ratio of the dataset and the dataset itself. In contrast, Zhang et al. [55] asserted that the splitting ratio is one of the primary issues. However, there is no general setting available as a solution. Based on their surveys, most researchers divide the datasets into lines with varying proportions of subsets. The most commonly used training and testing ratios are 90% to 10%, 80% to 20%, or 70% to 30%. As part of the training for this paper, the optimal prediction of the tuning parameters was achieved by dividing the actual data (96 samples) into 80% training and 20% test sets. To overcome the overfitting in RF, 30% of the training subset was used for the validation. A factorial combination of the tuning hyperparameters was selected to construct a grid and a search in that grid was performed in combination with a five-to-ten-fold cross-validation to achieve the best prediction performance based on the testing dataset [57]. After training and testing RF, the relative importance of the input variables was estimated using the optimal run.

The R statistical package software version 4.1.0 [58] was used for running the RF model and the multilayer perceptron neural network. R is a statistical programming language uniquely equipped to deal with different data. R makes it simple to manipulate data and generate publication-ready graphics and visualizations. The program can run all aspects of data analysis, mining, and modelling tasks. The *randomForest* package was used to run the RF model [59], the *caret* package was used for tuning the parameters [57], and the *iml* package was used to produce the accumulated local effect (ALE) plots [60]. For the multilayer perceptron neural network, *keras* and *tensorflow* packages were used in Python via R.

## 3. Results and Discussion

### 3.1. Experimental Results

**Figure 4:**
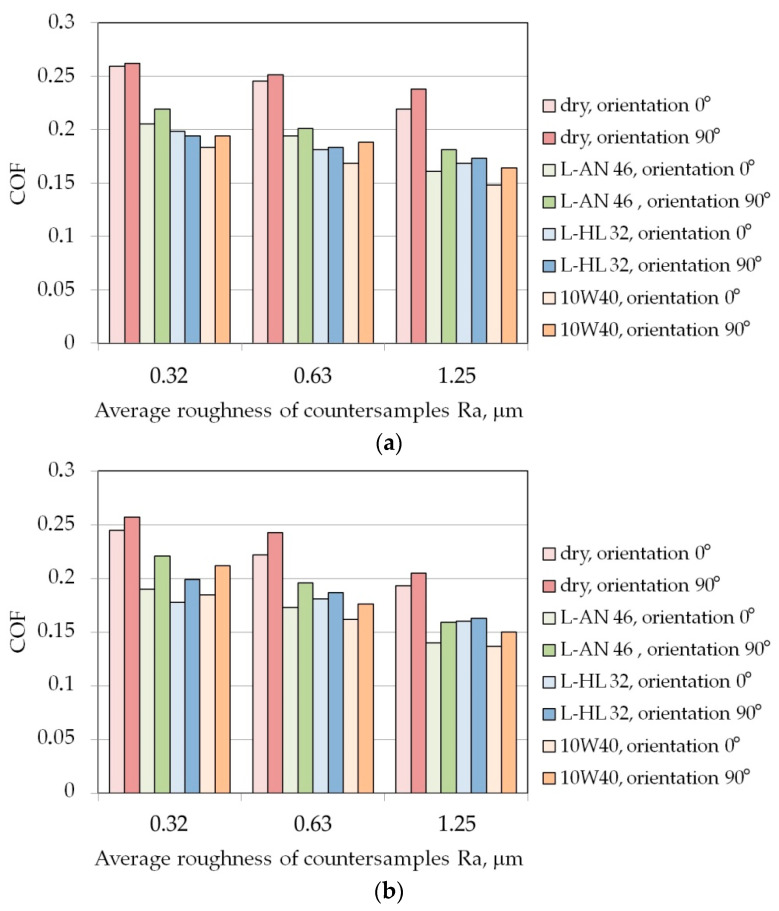

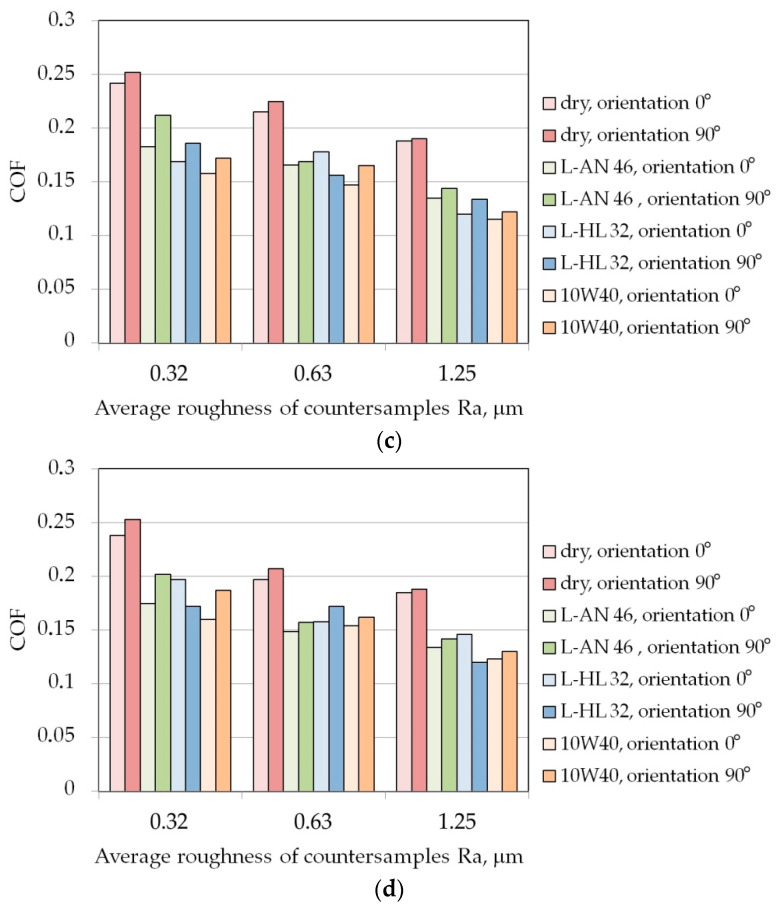
The effect of specimen orientation and friction conditions on the COF for (**a**) AW-5251-O, (**b**) AW-5251-H14, (**c**) AW-5251-H16, (**d**) AW-5251-H22.

In both the friction conditions tested, it was observed that the COF for samples cut along the sheet rolling direction was greater than for samples cut in the transverse direction (Figure 4). For the AW-5251-O aluminium alloy sheet, with the increase in the average roughness of the countersamples, the effect of the orientation of the samples on the value of the COF increases (Figure 4a). However, for the AW-5251-H22 aluminium alloy sheet, this relationship is reversed. In general, the greater the average roughness of the countersamples, the smaller the effect of the sample orientation on the COF. The relationships described above apply to both dry friction and oil lubrication. During the passage of the sample through the drawbead, the sheet is bent and straightened several times. As a result of the phenomenon of work hardening, the strength of the sheet material increases and, at the same time, the formability of the material decreases. Samples bent perpendicularly to the sheet rolling direction are less susceptible to plastic working than samples bent parallel to the rolling direction. This is due to the band-like directional arrangement of the material grains, which are elongated in the direction of rolling.

In general, increasing the average roughness of the countersamples causes a decrease in the value of the COF. The character of the influence of the surface roughness of a harder tool on the COF value compared to the sheet material depends on the friction conditions. During dry friction, the high surface roughness of the tool can lead to intensification of the ploughing phenomenon [61]. This is a particularly sensitive phenomenon in the case of aluminium alloy plates which are prone to galling. On the other hand, under lubricated conditions, increased surface roughness allows more oil to accumulate in the surface valleys [62]. The highest lubrication efficiency for both sample orientations was observed for 10W40 engine oil which is characterised by the highest viscosity index value among all the tested oils.

There is a clear tendency for the COF value to decrease with the increase in the average roughness of the countersamples. The sheet metal undergoes severe work hardening when passing through the drawbead and its hardness increases. In this way, the intensification of the ploughing phenomenon is limited. Therefore, the increased surface roughness of the strip sample ensures that more lubricant is stored in the surface valleys [63].

Determining the influence of sheet surface roughness and mechanical properties on the value of the COF is extremely difficult due to the possible complex interactions of many parameters. Therefore, in the next section, machine learning and ANNs are used to identify the main interactions between the input parameters and the COF.

### 3.2. Artificial Neural Networks

**Figure 5:**
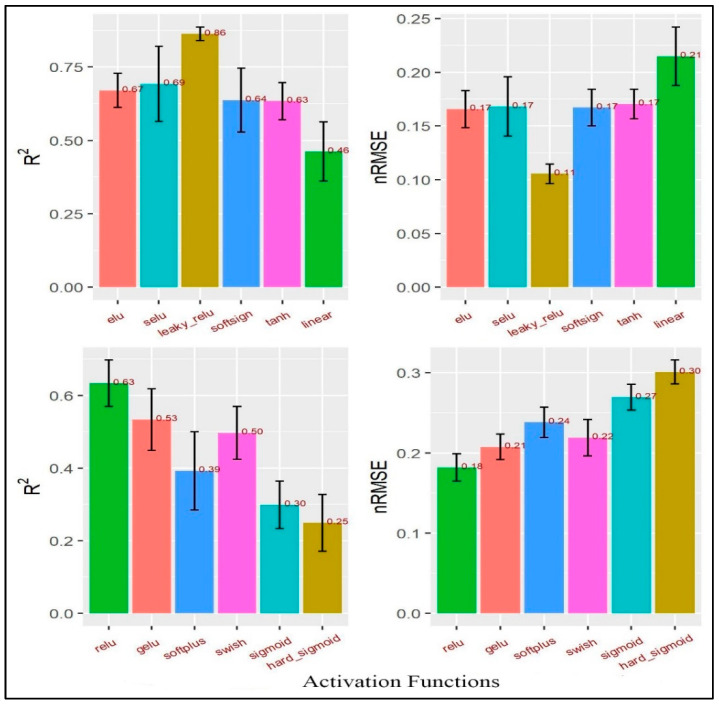
R^2^ and nRMSE in the MLP.

**Figure 6:**
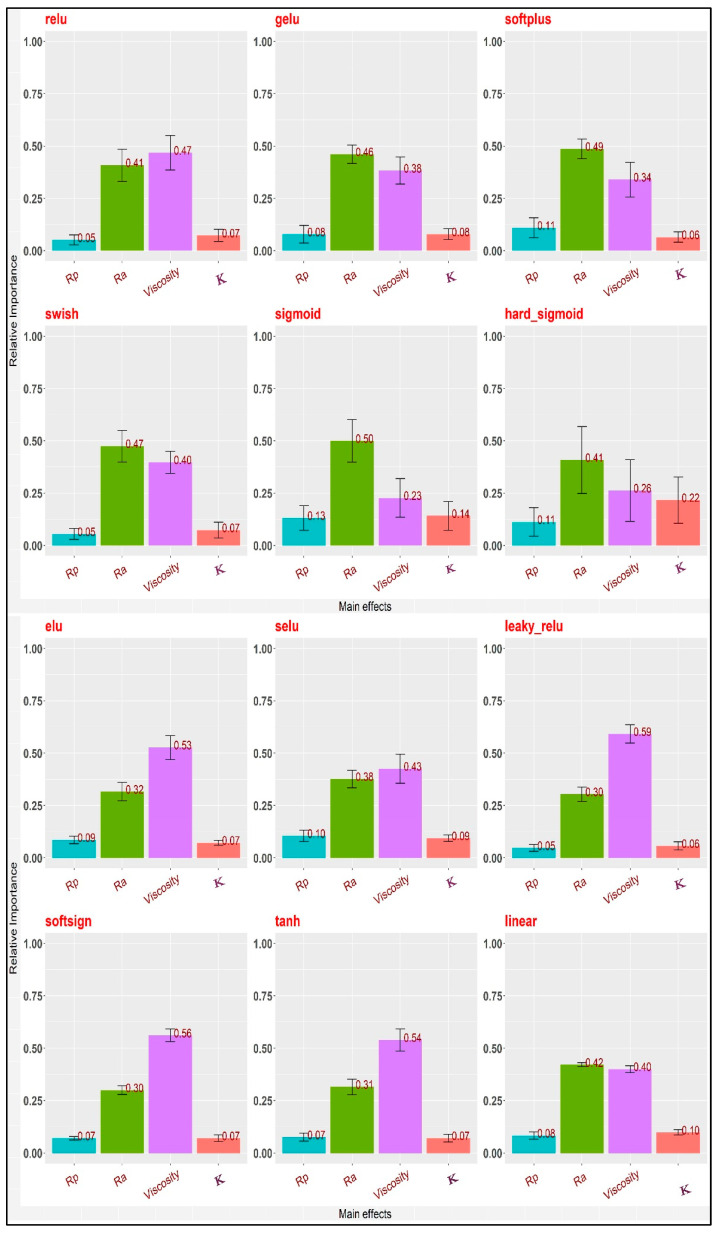
The relative importance of the inputs in the MLP.

When R^2^ is close to 1, the performance is good; when nRMSE is close to 0, the error is low. Knowing the difference between training and test errors is very important. Training errors are calculated using the same data for training the model. Test errors, on the other hand, are calculated using a complete dataset that was not used to train the model. It can be said that the R^2^ value of the training dataset shows how different the learned samples are, while the R^2^ value of the testing dataset shows how efficient the model is at making predictions. The *leaky_relu* activation function (Table 6) was the most suitable function for the tested data as its R^2^ value was the highest (0.86) and its nRMSE was the lowest (0.11) (Figure 5), so the estimated relative importance of the inputs based on that activation function was more reliable than the other activation functions. The importance of an input was measured by calculating the increase in the prediction error of the model after permuting the input. An input is considered important if shuffling its values caused an increase in the model error because, in this situation, the model relied on that input for its prediction. On the other hand, an input is considered unimportant if shuffling its values caused no change in the model error because, in this state, the model disregarded the input. Oil viscosity was the most important input to the COF (0.59) followed by the average roughness of the countersamples Ra (0.30), while both the yield stress R_p0.2_ and the strength coefficient K were the least important inputs (0.05 and 0.06, respectively). The hard sigmoid activation function was the worst function as its R^2^ value was the lowest (0.25) and its nRMSE value was the highest (0.30) (Figure 6), so it should not be used. 

From Figure 6, it is clear that the results of the different activation functions varied greatly. Predicting a COF with the same features using various activation functions might be problematic. Activation functions may utilise these features well or inadequately. Not all prediction models can leverage data links for predicting the COF. The issue is stochastic; the data set is incomplete, the data are inadequate, the model is too simplistic, or any combination of these. All the foregoing concerns would lead to discrepancies in unseen data model predictions. To estimate the relative importance of the inputs, the Glorot uniform initialiser [$\sqrt{6/\left(\mathrm{fan}\_\mathrm{in}\;+\;\mathrm{fan}\_\mathrm{out}\right)}$] where fan_in is the number of input units and fan_out is the number of output units] was used to generate the connection weights to avoid gradient vanishing problems in the deep learning and the connection weight algorithm, (${\mathrm{RI}}_{x}=\sum_{y=1}^{m}w_{\mathrm{xy}}w_{\mathrm{yz}}$) where *W_xy_* is the connection weights from the input layer to the hidden layer and, *W_yz_* is the connection weights from the hidden layer to the output layer. The relative importance of the inputs was calculated using 12 different activation functions (Figure 6).

**Figure 7:**
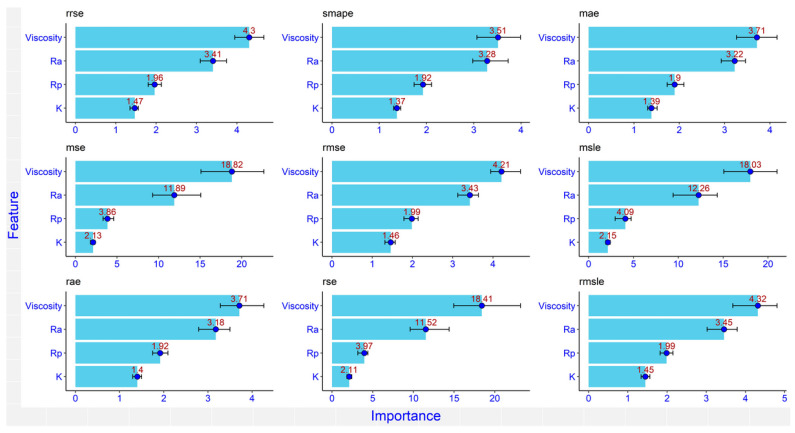
The relative importance of the inputs in the RF model using nine different loss functions.

On the other hand, variable importance (Figure 7) was estimated using the optimal run after training and testing the RF model (R^2^ = 0.90 ± 0.028) using nine different loss functions (Table 7). The dots represent the median of the relative importance while the lines represent the minimum and maximum values of the relative importance. The function of variable importance in the RF model defines the insight of the additive predictive value of a certain input variable and consequently ranks the studied input variables by using the mean decrease in accuracy that happened when a particular input variable was permuted randomly, where the mean decrease in accuracy is an indication of how much accuracy was lost in the prediction process by removing each input variable. Input variables with a big mean decrease in accuracy are considered the most important or strongest input variable to the output.

Before estimating the importance of the input variables, it is worth noting that when collinearity among inputs is present, the estimated variable importance becomes less reliable and less interpretable and, consequently, ALE plots should be used to explore the nature of the relationship between the inputs and the output. However, when collinearity is absent, variable importance becomes reliable and, consequently, partial dependence (PD) plots could be used for providing information about the shape of the relation between each input and the output [64].

**Figure 8:**
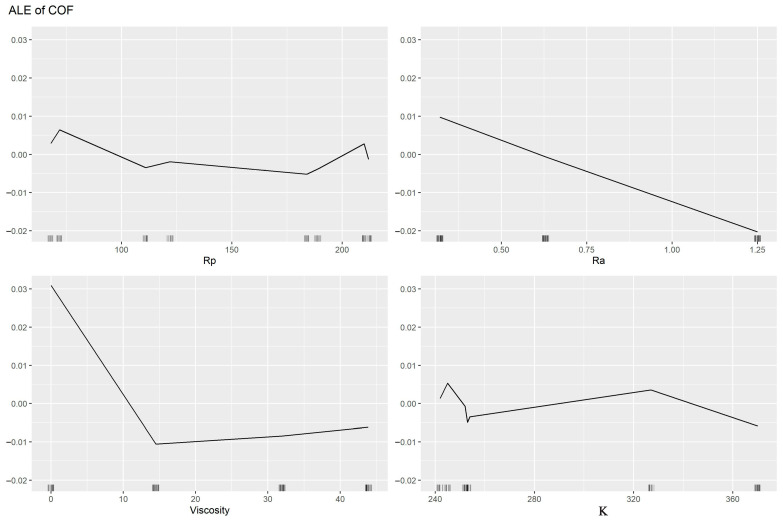
ALE plots showing the effect of the studied inputs on predicting the COF.

Figure 8 depicts the influence of the analysed inputs on predicting the COF using ALE plots. 

ALE plots illustrate how the prediction average of the output changes by changing the values of an input while all other inputs are kept at their original values. Figure 8 reveals that oil viscosity and Ra were the most important inputs to the COF, while R_p0.2_ and K were the least important. These results are in agreement with those obtained by the results of the ANNs. The most essential factor influencing friction conditions is viscosity. The surface roughness does not determine the volume of oil retained and thereby affects the COF values. Roughness is critical in increasing friction. Moreover, if roughness retains oil, then there is a correlation between the oil viscosity and the roughness input parameters in determining the output COF. As R_p0.2_ and K were the minor relevant inputs, it may be deduced that the mechanical characteristics of the sheets did not make a substantial contribution. There was no noticeable increase in the intensity of the mechanical interactions occurring on the surface asperities.

**Figure 9:**
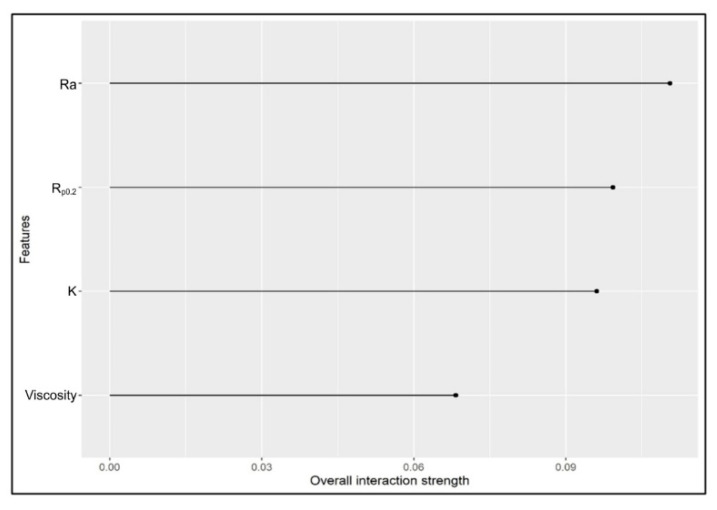
Overall interaction strength of the studied parameters (the scale of the interaction is 0 to 1).

**Figure 10:**
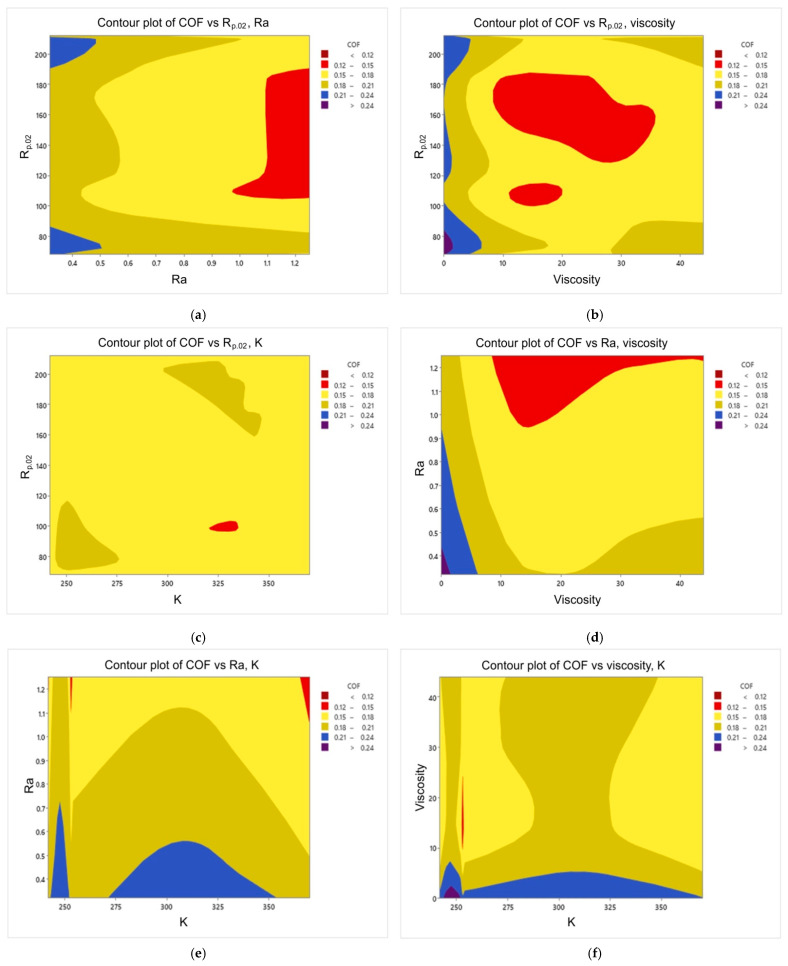
Contour plots of the studied inputs in relation to the output: (**a**) COF vs. R_p0.2_ and Ra, (**b**) COF vs. R_p0.2_ and oil viscosity, (**c**) COF vs. R_p0.2_ and K (strength coefficient), (**d**) COF vs. Ra and oil viscosity, (**e**) COF vs. Ra and K, (**f**) COF vs. oil viscosity and K. Red colour means low values of COF.

Figure 9 and Figure 10 reveal that the Ra and R_p0.2_ were the most active inputs in interactions with the other inputs. Viscosity was the lowest in interactions with the other inputs because it has a large direct effect. However, the Ra has both a large direct effect and higher interactions with the other inputs. Figure 10 shows that when the Ra and R_p0.2_ interacted with each other this leads to a lower COF (coloured red) when the Ra values were more than 1.1 and the R_p0.2_ values between 105 and 190. When the oil viscosity is added with the Rp, the values of the COF were lower at 9–35 for viscosity and 105–190 for R_p0.2_ with a gap between 110 and 130. The interaction between the oil viscosity and the Ra revealed a low value of the COF when the oil viscosity values were between 9 and 35 and the Ra values between 0.95 and 1.25. On the other hand, the interaction between K and the other inputs was the least important one because it leads to a very small region of lower values of the COF (coloured red). It can be concluded that to achieve lower values of the COF, the R_p0.2_ values should be between 105 and 190, the Ra values should be between 0.95 and 1.25, and the oil viscosity values should be between 9 and 35. It is highly challenging to explain the interplay of the many parameters physically. It can be observed that there is an unsteadiness at K = 255 MPa for both Ra and viscosity; this is because it is not necessary to be able to compute the accumulated local effects (ALE) since there was no connection between the inputs, that is, the changes in predictions did not average out significantly across the grid. The Y axis represents the deviation from the predicted value to the observed value. Therefore, once the viscosity was over 10, the difference was negligible.

## 4. Conclusions

In this paper, the results of the frictional performance of AW-5251 aluminium alloys sheet in various tempers were tested using a drawbead simulator. The RF machine learning algorithm and ANNs were used to identify the parameters affecting the COF. Based on the experimental results and ANN modelling, the following conclusions can be drawn:

- It was observed that the COF for samples cut along the sheet rolling direction was greater than for samples cut in the transverse direction. This applies to both dry friction and lubricated conditions. For the AW-5251-O sheet, the greatest difference (0.019) in the COF values for both sample orientations was observed for dry friction conditions for a countersample with an average roughness of 1.25 µm. For the AW-5251-H14 sheet, the greatest difference (0.021) in COF values for both sample orientations was observed for dry friction conditions for a countersample with an average roughness of 0.63 µm.
- In general, the greater the average roughness of the countersamples, the smaller the effect of sample orientation on the COF.
- There is a clear tendency for the COF value to decrease with the increase in the average roughness of the countersamples. Increasing the surface roughness of the countersample material with much greater strength than the workpiece material causes intensification of the mechanical interaction of the surface asperities, but at the same time, greater roughness means a larger volume of the valleys constituting the lubricant reservoir.
- The highest lubrication efficiency for both sample orientations was observed for SAE10W40 engine oil which is characterised by the highest viscosity index value (157) among all the tested oils.
- Oil viscosity was the most important input to the COF followed by the average roughness of the countersamples Ra, while both R_p0.2_ and K (strength coefficient) were the least important inputs. As R_p0.2_ and K were the minor relevant inputs, it may be deduced that the mechanical characteristics of the sheets did not make a substantial contribution to the COF when passing the sheet metal through the drawbead.
- The most appropriate activation function for our data was *leaky_relu* because it had the highest R^2^ and the lowest nRMSE.
- The average roughness of the countersamples Ra and the yield stress R_p0.2_ were the most active inputs in interactions with the other inputs. Oil viscosity was the lowest in interactions with the other inputs because it has a large direct effect. However, the Ra has both a large direct effect and higher interactions with the other inputs.

## Figures and Tables

**Table 1:** Temper designations of the test materials.

Temper Type	Description
O	soft
H14	work hardened to half hard, nor annealed after rolling
H16	work hardened to three-quarter hard, nor annealed after rolling
H22	strain-hardened and partially annealed—three-quarter hard

**Table 2:** Chemical composition (wt.%) of the AW-5251 aluminium alloy according to the EN 573-3 standard.

Mn	Cu (Max.)	Mg	Si (Max.)	Zn (Max.)	Cr (Max.)	Ti (Max.)	Others (Total)	Fe (Max.)	Al
0.10–0.50	0.15	1.70–2.40	0.40	0.15	0.15	0.15	0.15	0.50	balance

**Table 3:** Basic mechanical properties of AW-5251 aluminium alloy sheets.

Temper Type	Specimen Orientation, °	Elongation A_50_	Ultimate Tensile Stress R_m_, MPa	Yield Stress R_p0.2_, MPa	Strength Coefficient K, MPa	Strain Hardening Exponent n
O	0	0.18	203	68	252	0.607
	90	0.25	205	72	245	0.870
H14	0	0.04	234	212	254	0.478
	90	0.04	241	210	327	0.786
H16	0	0.05	232	184	253	0.528
	90	0.06	236	189	242	0.751
H22	0	0.19	201	111	370	0.535
	90	0.21	207	122	370	0.793

**Table 4:** Basic surface roughness parameters of AW-5251 aluminium alloy sheets.

Temper Type	Sa, µm	Sv, µm	Sp, µm	Sku	Ssk
O	0.302	1.39	0.37	3.48	0.267
H14	0.340	1.62	2.48	3.34	0.298
H16	0.362	2.08	2.98	3.67	0.338
H22	0.325	1.53	2.04	3.58	0.321

**Table 5:** Physico-chemical properties of the test oils.

Oil Type	Kinematic Viscosity, mm^2^/s	Viscosity Index
L-AN 46	43.90	94.0
L-HL 32	32.00	95.0
SAE 10W40	14.50	157.0

**Table 6:** Mathematical model of the 12 activation functions used.

Name of Activation Function	Mathematical Equation
Rectified linear unit (ReLU)	*f*(*x)* = *max* (*x*,0)
Gaussian Error Linear Unit (GELU)	*f*(*x*) = *x* * *P* (*X* ≤ *x*)
Softplus	*f*(*x*) = *ln* (1 + *e^x^*)
Sigmoid-Weighted Linear Unit (Swish)	*f*(*x*) = *x*/(1 + *exp*(−*x*))
Sigmoid	*f*(*x*) = 1/(1 *+ e^−x^*)
Hard sigmoid	*f*(*x*) = *max* (*min* (0:25*x* + 0:5;1);0)
Exponential linear unit (ELU)	*ifelse* (*x <* 0,1.673263 * (*exp*(*x*) *−* 1.0507), *x*)
Scaled exponential linear unit (Selu)	*ifelse* (*x < =* 0,1.0507 * 1.673263 * (*exp*(*x*) *−* 1.0507), *x* * 1.0507)
Leaky ReLU	*ifelse* (*x < =* 0, *α* * *x*, *x*) *where* 0 *< α <* 1
Sofsign	*f*(*x*) = *x*/(*|x| +* 1)
Tanh	*f*(*x*) = 2/(1 + *e*^−2*x*^) − 1
Linear	*f*(*x*) = *x*

**Table 7:** Mathematical model of the nine loss functions used.

Name of Loss Function	Abbreviation	Equation
root relative squared error	rrse	$\sqrt{\left[\overset{n}{\underset{i=1}{\sum }}{\left(x_{i}-y_{i}\right)}^{2}\right]/\left[\overset{n}{\underset{i=1}{\sum }}{\left(x_{i}-\tilde{x}\right)}^{2}\right]}$
symmetric mean absolute percent error	smape	$\frac{2}{n}\overset{n}{\underset{i=1}{\sum }}\frac{\left|x_{i}-y_{i}\right|}{\left|x_{i}\right|+\left|y_{i}\right|}$
mean absolute error	mae	$\frac{1}{n}\overset{n}{\underset{i=1}{\sum }}\left|x_{i}-y_{i}\right|$
mean squared error	mse	$\frac{1}{n}\overset{n}{\underset{i=1}{\sum }}{\left(x_{i}-y_{i}\right)}^{2}$
root mean squared error	rmse	$\sqrt{\frac{1}{n}\overset{n}{\underset{i=1}{\sum }}{\left(x_{i}-y_{i}\right)}^{2}}$
mean squared log error	msle	$\frac{1}{n}\overset{n}{\underset{i=1}{\sum }}{\left(ln\left(1+x_{i}\right)-ln\left(1+y_{i}\right)\right)}^{2}$
relative absolute error	rae	$\left[\overset{n}{\underset{i=1}{\sum }}\left|x_{i}-y_{i}\right|\right]/\left[\overset{n}{\underset{i=1}{\sum }}\left|x_{i}-\tilde{x}\right|\right]$
relative squared error	rse	$\left[\overset{n}{\underset{i=1}{\sum }}{\left(x_{i}-y_{i}\right)}^{2}\right]/\left[\overset{n}{\underset{i=1}{\sum }}{\left(x_{i}-\tilde{x}\right)}^{2}\right]$
root mean squared log error	rmsle	$\sqrt{\frac{1}{n}\overset{n}{\underset{i=1}{\sum }}{\left(ln\left(1+x_{i}\right)-ln\left(1+y_{i}\right)\right)}^{2}}$

## Data Availability

Data is contained within the article.
